# Berberis lyceum root bark extract attenuates anticancer drugs induced neurotoxicityand cardiotoxicity in rats

**DOI:** 10.4314/ahs.v22i3.22

**Published:** 2022-09

**Authors:** Sidra Qurat-ul-Ain, Anwar Rukhsana, Sahar Isma Tariq, Ashiq Kanwal

**Affiliations:** 1 University College of Pharmacy, University of the Punjab Lahore, Pakisatn; 2 University of the Punjab, University College of Pharmacy, University of the Punjab, Lahore, Pakistan; 3 Superior University, Faculty of Pharmaceutical Sciences Superior College, Superior University 17-km Raiwind Road Lahore, Pakistan

**Keywords:** Doxorubicin, Cisplatin, Berberis lyceum, Cardiotoxicity, Neurotoxicity, Antioxidant

## Abstract

**Background:**

Traditionally, *Berberis lyceum* was extensively used for the treatment of several human diseases.

**Objective:**

This study was undertaken to determine in vivo effects of Berberis lyceum root bark against doxorubicin-induced cardiotoxicity and cisplatin-induced neurotoxicity in Sprague Dawley rats.

**Methods:**

A single dose of doxorubicin (20 mg/ kg i. p) and cisplatin (4mg/kg i.p) was used to induce cardiotoxicity and neurotoxicity, respectively. Berberis lyceum methanolic extract was given orally (200 and 400 mg/ kg) to toxicity-induced rats. The cardiac biomarkers i.e. serum aspartate aminotransferase, alanine transaminase, lactate dehydrogenase, creatine kinase and creatine kinase MB were analyzed in blood collected from cardiotoxic rats. The tissue oxidative stress markers included protein, glutathione s-transferase specific activity, catalase activity, total glutathione, and malondialdehyde levels were measured in cardiac and brain homogenate of the respective groups.

**Results:**

Berberis lyceum methanolic extract has the potential to reduce the doxorubicin-induced cardiotoxicity and cisplatin-induced neurotoxicity significantly (*p<0.05) by reducing the serum markers and oxidative stress parameters. Histopathological analysis exhibited a marked improvement in the morphology of cardiac and brain tissues.

**Conclusion:**

It is concluded that methanolic extract of Berberis lyceum root bark has the potential to protect and reverse anticancer drugs induced cardiotoxicity and neurotoxicity.

## Introduction

Cancer is a neoplastic disorder that is a chief cause of death in many developed and emerging countries. The global economic burden of cancer is substantially raised and due to this disease, 7.6 million (13%) deaths are reported annually. For many years, medicinal plants have been used to cure or prevent a diverse range of disorders. There is a sufficient number of scientific literature is available that verified the anticancer potential of medicinal plants due to the presence of various bioactive chemicals.^1^ Chemoprevention is defined as either avoid or undo the toxic effect (neoplasia genesis) of a chemical by using another chemical agent. It is documented that the plant bioactive compounds provide protection against tumor genesis by several mechanisms such as instigation of apoptosis, reducing free radical production and oxidative stress, inhibition of cell proliferation, and interrupting the cancer cell cycle process. In previous studies, it is acknowledged that the concomitant use of antioxidant phytochemicals during chemotherapy can protect against anticancer drugs-related side effects.^2^

Doxorubicin (DOX) is a highly effective drug used to treat cancers like leukemia^3^, connective tissue cancers^4^, breast cancer 5, and lung cancer 6. However, its application is limited due to its cardiac targeted toxicity. Doxorubicin can induce cardiotoxicity by several mechanisms, including free radical generation, calcium excess, dysfunctional mitochondria, a mutation in gene expression, apoptosis, and causing defects in the natural immune system.^7^ Despite multifactorial doxorubicin-induced cardiotoxicity, oxidative stress plays a vital role to instigate cardiac injury. Doxorubicin produces oxidative stress by the generation of free radicals in the body. These free radicals are generated by the addition of electrons to doxorubicin quinone moiety, which rapidly regenerates to its parent structure by reducing oxygen to the superoxide anion and hydrogen peroxide (H2O2).^8^,^9^ The dismutation of the superoxide is catalyzed by acidic pH or superoxide dismutase (SOD) enzyme. As H2O2 is a less toxic molecule, it is eliminated by an enzymatic antioxidative defense system consisting of catalase and glutathione peroxidase.^10^ Further, a secondary metabolite called doxorubicinol which is formed by the reduction of the carbonyl group of doxorubicin also promotes heart toxicity.^11^

Cisplatin is a platinum-based chemical and widely used to cure various malignancies. It exerts its antineoplastic effects by the formation of DNA adducts, by induction of oxidative stress, and by triggering apoptosis in tumor cells. Cisplatin being an inert molecule requires activation before interacting with nucleophilic target sites. A non-enzymatic water-based reaction activates cisplatin into a charged molecule.^12^ Mitochondria are primary targets for cisplatin-induced toxicity. Mitochondrial DNA adducts are formed upon the interaction of activated cisplatin with mitochondrial DNA, resulting in direct damage, i.e. reduced protein synthesis, impaired electron transport chain function, oxidative stress, and activation of intracellular apoptosis.^13^ Neurotoxicity is the onerous side effect associated with cisplatin use. Cisplatin possibly causes neurotoxicity due to many reasons such as oxidative stress, deposition of cisplatin adduct in neurons, and inhibition of DNA repairing mechanisms mainly in peripheral neurons and dorsal root ganglia neurons. This menacing side effect leads to either cisplatin dose reduction or early discontinuation of therapy that can badly affect the patient's life.^14^

Berberis lyceum Royle belongs to the family Berberidaceae and it is a common medicinal plant found in Pakistan. Berberis lyceum fruit is known as “Kashmal” while the root is called “Darhald”.^15^ Traditionally, Berberis lyceum was extensively used for the treatment of several human diseases. The literature review reveals that it possesses antihyperlipidemic 16, anti-diabetic 17, antimicrobial, antifungal^18^, antioxidant and nephron-protective activity.^19^ Berberis lyceum contains valuable phytochemicals, i.e. alkaloids (berberine, berbamine, palmitine), hydrolysable tannins, cardioactive glycosides, and saponins which are responsible for its various pharmacological effects.^20^

The study aimed to evaluate the protective effects of Berberis lyceum root bark against doxorubicin-induced cardiotoxicity and cisplatin-induced neurotoxicity in Sprague Dawley rats because previous no such study was found through an extensive literature survey.

## Materials And Methods

### Chemicals

Doxorubicin (Brand name: Adrim) intravenous infusion was purchased from Atco Pharma Laboratories (Pvt). Ltd. Anthrone reagent was obtained from Sigma life sciences, Germany. Folin and Ciocalteu's phenol reagent and Triton X acquired from Unichem chemicals, Ireland. Vitamin E was procured from Merck Pvt. Ltd. For aspartate transaminase (AST) and alanine transaminase (ALT) analysis, kits by Singapore Biosciences (SBio) PTE Ltd. were used. The kits by Randox Laboratories Ltd. were used to estimate lactate dehydrogenase (LDH), creatine kinase (CK), and creatine kinase myocardial band (CKMB). All chemicals were analytical grade.

### Extraction of Berberis lyceum Royle root bark

Berberis lyceum dried root bark was powdered. 250 g of powder was soaked in 800 mL of methanol and macerated for 6 days with intermittent stirring. This mixture was filtered and residues were again soaked in methanol for 3 days. The filtrate was pooled and dried by a rotary evaporator at 45°C. A total of 25 g of Berberis lyceum methanol extract (MEBL) was obtained. The extract was stored at 4 – 8°C.

### Chemical composition of Berberis lyceum methanolic extract

Qualitative phytochemical screening of Berberis lyceum methanolic extract was performed to detect the presence of various chemical compounds such as glycosides, saponins, tannins, alkaloids, flavonoids, triterpenoids, carbohydrates, proteins, fats and, fixed oils.

The quantitative analysis of the phytochemicals (primary and secondary metabolites) was also performed according to the standard procedures. Primary metabolites estimation was included total protein 21, total lipids 22, and total carbohydrates 23. Secondary metabolites were also determined such as total polyphenols 24, total flavonids 25, total glycosaponins and, total polysaccharides.^26^

### Animals

Sprague Dawley rats were procured from the University of Health Sciences (UHS) Lahore. The animals were handled as per guidelines of an Animal Ethics Committee of University College of Pharmacy (PUCP), University of the Punjab Lahore, Pakistan. The committee reviewed and approved the experiment protocol (issued voucher no: AEC/PUCP/1051). The rats were acclimatized and maintained under controlled conditions; temperature 25°C ± 2 and 60% relative humidity with a 12/12 hour dark and light cycle. Animals were provided a standard diet and water ad libitum.

### Experimental protocol

For the in vivo study, animals were randomly selected from the animal house. The extract was dissolved in distilled water (DW) and vortexed to get a homogenous mixture. Animals were grouped as the following scheme having 6 rats in each group:

GROUP 1 (Control): Rats were given water as a vehicle for 21 days.

GROUP 2 (DOX treated): Animals were given vehicle for 21 days and on the 19th day, a single dose of doxorubicin 20 mg/ kg body weight (BW) was administered intraperitoneally.

GROUP 3 (MEBL-200): Animals were administered Berberis lyceum methanol extract 200 mg/ kg BW through oral gavage for 21 days.

GROUP 4 (MEBL-400): Animals were administered 400 mg/ kg BW of Berberis lyceum methanol extract through oral gavage for 21 days.

GROUP 5 (DOX+MEBL-200): Rats were administered 200 mg/kg BW of Berberis lyceum methanol extract through oral gavage for 21 days. On the 19th day, a single injection of doxorubicin 20 mg/ kg was co-administered intraperitoneally.

GROUP 6 (DOX+MEBL-400): Animals were administered 400 mg/ kg BW of Berberis lyceum methanol extract through oral gavage for 21 days. On the 19th day, a single injection of doxorubicin 20 mg/ kg BW was co-administered intraperitoneally.

GROUP 7 (DOX+Vitamin E): Group was administered vitamin E 100 mg/ kg BW through oral gavage for 21 days. On the 19th day, a single injection of doxorubicin 20 mg/ kg BW was co-administered intraperitoneally

GROUP 8 (Cisplatin treated): Animals were given vehicle for 21 days and on the 19th day, a single dose of cisplatin 4 mg/ kg BW was administered intraperitoneally.

GROUP 9 (Cisplatin+MEBL-200): Rats were administered 200 mg/kg BW of Berberis lyceum methanol extract through oral gavage for 21 days. On the 19th day, a single injection of cisplatin 4mg/ kg BW was co-administered intraperitoneally.

GROUP 10 (Cisplatin+MEBL-400): Animals were administered 400 mg/ kg BW of Berberis lyceum methanol extract through oral gavage for 21 days. On the 19th day, a single injection of cisplatin 4 mg/ kg BW was co-administered intraperitoneally.

Blood samples from each rat were taken after 24 hours of the last dose of doxorubicin and cisplatin. All the rats were sacrificed and the heart from cardiotoxic and brain from neurotoxic group rats was removed. A small section of the heart and brain from each group were preserved in 10% formalin for histopathological examination. All the tissue samples were stored at -80°C for further analysis.

### Preparation of blood serum

The blood samples from rats were taken by cardiac puncture and collected in the serum gel separating tube. Then these samples were allowed to stand at 25°C for 30 minutes and subsequently centrifuged at 3000 rpm for about 15 minutes. The clear serum was collected from the gel top while cell debris settled down in the gel. Serum was transferred to labeled tubes and refrigerated at -80°C for future experimentation.

Preparation of heart homogenate and brain homogenate Pooled hearts were finely chopped in phosphate buffer through scissors. Heart tissues were then homogenized with the help of a homogenizer then centrifuged at 12,500 × g at 4°C for 20 minutes. The final post mitochondrial supernatant (PMS) was carefully transferred to Eppendorf 's tubes and stored at -80°C for further analysis.

### Estimation of biochemical parameters

The serum biochemical markers in cardiotoxic groups included aspartate transaminase (AST), creatine kinase (CK), alanine transaminase (ALT), lactate dehydrogenase (LDH) , creatine kinase MB (CK-MB) were measured according to the procedure specified within the kits. The Lowry method was applied for the estimation of total protein in PMS.21

### Glutathione s-transferase (GST) assay

The GST-specific activity was estimated by Habig's method with slight modification. For GST-specific activity, the reaction mixture contained post mitochondrial supernatant of brain and heart, 100 mM phosphate buffer (pH 6.5), 30 mM GSH, and 30 mM CDNB. Change in the absorbance was measured at 340 nm on a spectrophotometer for 5 minutes. GST activity expressed in µmol/minute/mg protein.^27^

### Glutathione (tGSH) assay

Total GSH content was determined by Sedlak and Lindsay method with slight modification. Heart and brain tissues were homogenized in 67 mM phosphate buffer (pH 7.4) separately. 25% trichloroacetic acid added to each homogenate and centrifuged at 4,200 rpm for 40 minutes. Afterward, supernatants of each sample were separated. 200 mM Tris HCl buffer containing 0.2 M EDTA (pH 7.5), 10 mM DTNB, and methanol added to each supernatant. Then, the reaction mixture was incubated for 30 minutes at 37°C. The absorbance of the final yellow solution was recorded on a spectrophotometer at wavelength 412 nm.^28^

### Catalase analysis for heart and brain tissues

Catalase activity measured according to Sinha prescribed method with minor modification. The tissue homogenate (heart/ brain) was vortexed with 0.01 M phosphate buffer (pH 7) and freshly prepared 0.2 M H2O2 Dichromate/acetic acid added to the reaction mixture. Then, this reaction mixture heated for 10 minutes till permanent green color appeared. The absorbance was taken at 570 nm at room temperature, and catalase activity was measured directly from the standard curve.^29^

### Malondialdehyde (MDA) assay

Malondialdehyde (MDA) test was carried out by using thiobarbituric (TBA) assay followed by Ohkawa method with a slight alteration in the experimental steps. Heart and brain tissue samples (1mg) were homogenized in 1.2% KCl separately. To homogenate, 20% acetic acid, 8% sodium lauryl sulfate, 20.8% TBA, and distilled water were added. The reaction mixture was incubated for 1 hour at 98°C. After incubation, butanol: pyridine (15:1) was added to the mixture and centrifuged for 30 min (4,000 rpm). The absorbance of the resultant supernatant was recorded at 532nm. The concentration of MDA (mM/g tissue) was directly calculated from the MDA standard curve.^30^

### Histopathological studies

10% formalin solution was used to fix each heart and brain tissue. The specimens were handled as per standard procedure and embedded in paraffin wax. The blocks were sectioned and stained using the hematoxylin-eosin (H & E) method and examined by light microscopy.^31^

### Statistical analysis

Graph Pad Prism version 7.01 software was used for the statistical computation of results. Analysis was done by using unpaired t-test, one-way ANOVA, and by Dunnett's test. All data expressed in mean standard deviation (Mean ± SD) and *P <0.05 was considered statistically significant.

## Results

The effect of Berberis lyceum on doxorubicin-induced cardiotoxicity and cisplatin-induced neurotoxicity in rats was estimated by measuring serum markers, tissue markers, and by histopathology of the heart and brain tissues. The general appearance of all groups of animals was observed and noted throughout the study. In the doxorubicin treated group, there was drastic weight loss and fur had a pink tinge. Rats had soft watery feces and red exudates also appeared on the sides of the eyes and nose. These observations were significantly less in concurrent administration therapy (doxorubicin and Berberis lyceum treated group).

### Chemical composition of Berberis lyceum methanolic extract

Berberis lyceum qualitative and quantitative phytochemical screening (Table 1 and Table 2) revealed that plant root contains many important primary and secondary metabolites, i.e. proteins, lipids, carbohydrates, alkaloids, tannins, saponins, flavonoids, and polyphenols.

**Table 1:** Qualitative estimation of the chemical composition of Berberis lyceum methanolic extract

Phytochemical	Name of test(s)	Results
Glycosides	Keller-Killiani test	Positive
Saponins	Foam test	Positive
Tannins	Ferric chloride test	Positive
Alkaloids	Dragendroff's test Hager's test Mayer's test Wagner's test	Positive
Flavonoids	Lead acetate test Alkaline reagent test	Positive
Triterpenoids,	Salkowaski test Libermann burchard Test	Positive
Carbohydrates,	Molisch's test Fehling's test Seliwanoff's test	Positive
Amino acids	Ninhydrin test	Negative
Fats and fixed oils	Saponification test	Positive

**Table 2:** Quantitative estimation of the chemical composition of Berberis lyceum methanolic extract

**Primary** **metabolites**	**Total proteins (%)**		**Total lipids (%)**	**Total** **carbohydrates** **(%)**
	0.8±0.6		2.6±0.2	94.2±0.5

**Secondary** **metabolites**	**Total** **Polyphenols** **(mg/g)**	**Total Flavonoids** **(mg/g)**	**Total** **Glycosaponins** **(mg/g)**	**Total** **Polysaccharides** **(mg/g)**
	78.84±0.03	118.83 ± 0.06	16.9±0.05	132.56±0.02

### Effect of Berberis lyceum methanolic extract on serum AST (U/L)

The study outcomes showed that serum AST level in the doxorubicin treated group was significantly (*p < 0.05) increased (240.66 ± 8.32 U/L) than the control group (148.37 ± 11.5 U/L). The methanolic extract groups (MEBL-200 and MEBL-400) demonstrated an insignificant change in the AST level against the control. The concurrent administration of the doxorubicin and Berberis lyceum methanolic extract significantly decreased the AST level as compared to the doxorubicin treated group. For group 5 and group 6, a decrease in the AST level: 166.03 ± 8.58 U/L and 150.21 ± 4.49 U/L was reckoned, respectively. The co-administration of the doxorubicin and vitamin E also showed a significant drop (152.47 ± 8.01 U/L) in the AST level (Figure 1A).

**Figure 1:**
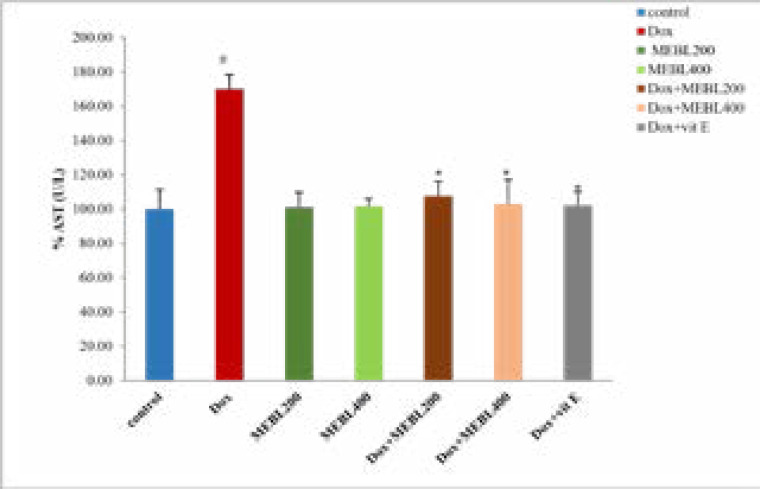
Effect of Berberis lyceum methanolic extract: serum AST (A), serum ALT (B), Creatine kinase (C), serum CK-MB (D), LDH (E), PMS protein content (F) in doxorubicin-induced cardiotoxicity. The result analysis was conducted by using one-way ANOVA followed by Dunnett's test. Values expressed in Mean ± SD, (n=6) and p < 0.05 was considered significant.

### Effect of Berberis lyceum methanolic extract on serum ALT (U/L)

This study has exhibited that the ALT level in the doxorubicin treated group was significantly (*p < 0.05) increased (77.07 ± 2.8 U/L) than the control group (40.6 ± 4.83 U/L). The methanolic extract groups (MEBL-200 and MEBL-400) showed an insignificant change in ALT level as compared to the control. The co-administration of doxorubicin and Berberis lyceum methanolic extract significantly reduced the ALT level in comparison with the doxorubicin-treated group. For group 5 and group 6, a decrease in the ALT level: 50.77 ± 1.93 U/L and 43.7 ± 2.20 U/L was observed, respectively. The concurrent administration of the doxorubicin and vitamin E displayed a significant reduction (42.97 ± 2.54 U/L) in the ALT level (Figure 1B).

**Figure 1B:**
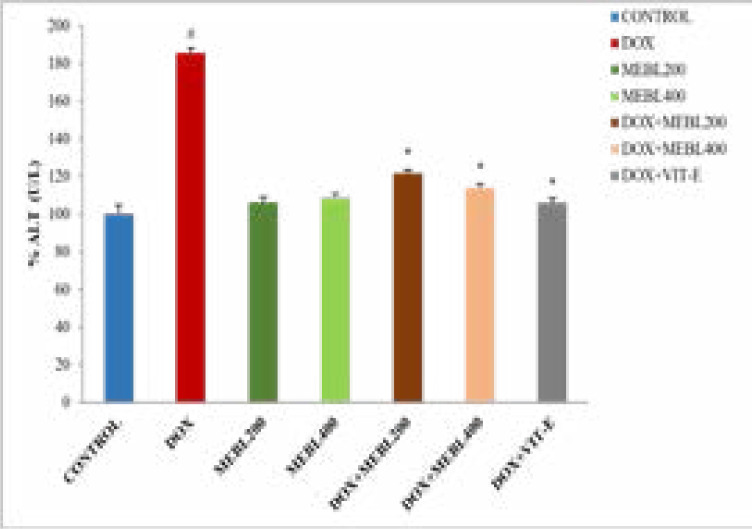


### Effect of Berberis lyceum methanolic extract on serum CK (U/L)

The present study results revealed that serum CK level in the doxorubicin treated group was significantly (#p < 0.05) increased (497.7 ± 8.91 U/L) than the control group (156.23 ± 5.26 U/L). The methanolic extract groups (MEBL-200 and MEBL-400) showed an insignificant change in serum CK level in comparison to the control. The concurrent administration of the doxorubicin and Berberis lyceum methanolic extract significantly decreased CK level against the doxorubicin-treated group. For group 5 and group 6, a decrease in the CK level: 223 ± 20.25 U/L and 206.2 ± 13.86 U/L was noticed, respectively. The co-administration of the doxorubicin and vitamin E also showed a significant decrease (213.47 ±16.3 U/L) in serum CK level (Figure 1C).

**Figure 1C:**
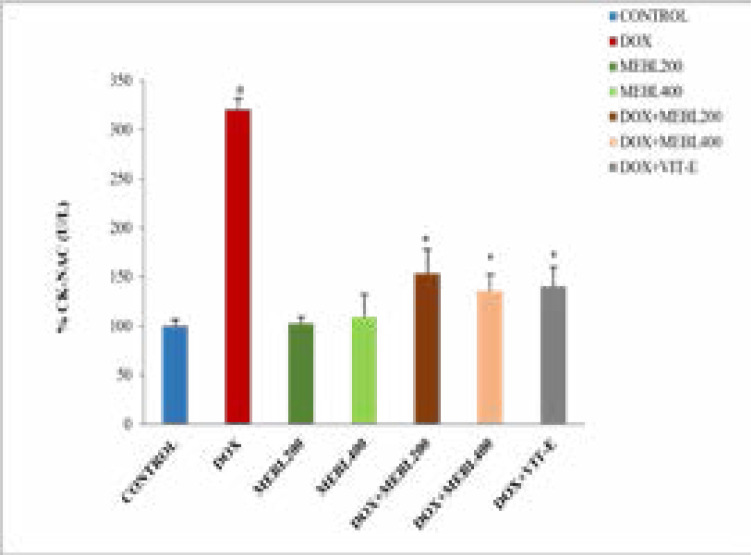


### Effect of Berberis lyceum methanolic extract on serum CK- MB (U/L)

The current investigation demonstrated that serum CK-MB level in the doxorubicin treated group was significantly (*p < 0.05) increased (206.16 ± 1.48 U/L) than the control group (65.93 ± 2.86 U/L). The methanolic extract groups (MEBL-200 and MEBL-400) showed an insignificant change in serum CK-MB level as compared to the control. The concomitant administration of the doxorubicin and Berberis lyceum methanolic extract significantly decreased the CK- MB level against the doxorubicin-treated group. For group 5 and group 6, a decrease in the CK-MB level: 140.73 ± 1.92 U/L and 116.1 ± 1.56 U/L was seen, respectively. The co-administration of the doxorubicin and vitamin E manifested a significant reduction (115.93 ±2.89 U/L) in serum CK-MB level as compared to the doxorubicin-treated group (Figure 1D).

**Figure 1D:**
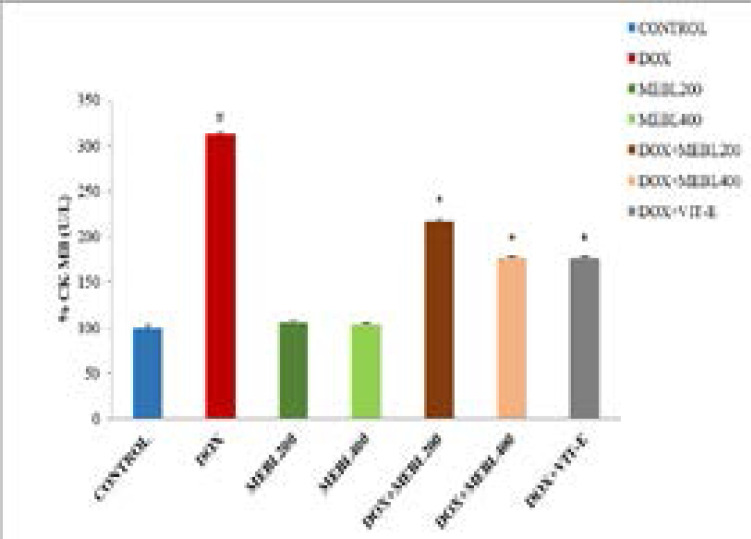


### Effect of Berberis lyceum methanolic extract on serum LDH (U/L)

This research outcome showed that serum LDH level in the doxorubicin-treated group was significantly (*p < 0.05) increased (837.43 ±8.44 U/L) than the control group (281.97 ± 23.2 U/L). The methanolic extract groups (MEBL-200 and MEBL-400) illustrated an insignificant change in serum LDH level contrasted with the control. The co-administration of doxorubicin and Berberis lyceum methanolic extract significantly decreased the LDH level as compared to the doxorubicin-treated group. For group 5 and group 6, a decrease in the LDH level: 588.5 ± 14.16 U/L and 395.27±14.70 U/L was observed, respectively. The concomitant use of the doxorubicin and vitamin E also exhibited a significant drop-off (381.07±14.69 U/L) in serum LDH level (Figure 1E).

**Figure 1E:**
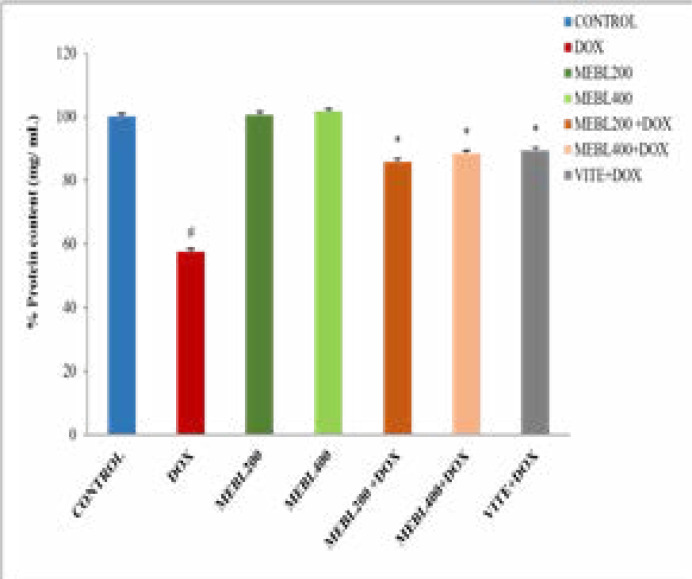


### Effect of Berberis lyceum methanolic extract on serum protein content (mg/mL)

The current study results showed that the protein content in the doxorubicin-treated group was significantly (# p < 0.05) decreased (39.13 ± 0.24 mg/mL) than the control group (68.07 ± 0.74 mg/mL). Berberis lyceum methanolic extract groups (MEBL-200 and MEBL-400) showed an insignificant change in protein content against the control. The parallel administration of the doxorubicin and Berberis lyceum methanolic extract significantly increased protein content over the doxorubicin-treated group. For group 5 and group 6, the respective increase in protein content was calculated as 68.43 ± 0.69 and 60.13 ± 0.25. The co-administration of the doxorubicin and vitamin E also showed a significant increase (60.80 ± 0.33 mg/mL) in protein content (Figure 1F).

**Figure 1F:**
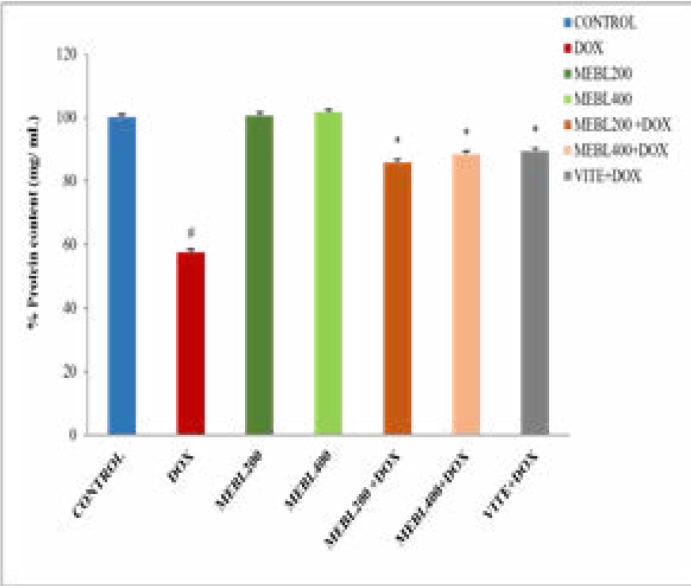


**Figure 2:**
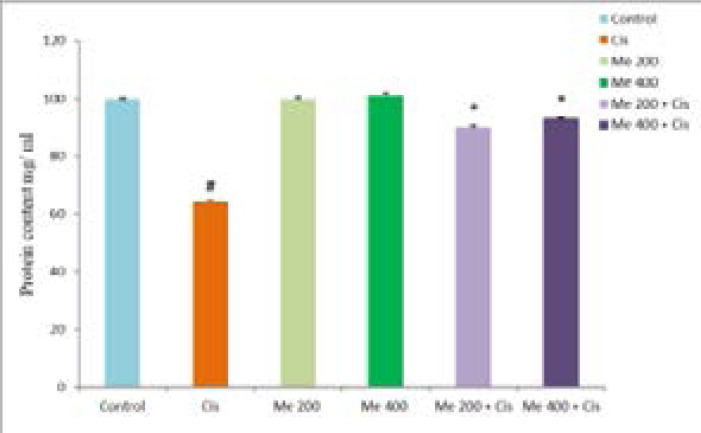
Effect of Berberis lyceum methanolic extract on PMS protein content in cisplatin-induced neurotoxicity. The result analysis was conducted by using one-way ANOVA followed by Dunnett's test. Values expressed in Mean ± SD, (n=6) and p < 0.05 was considered significant

The protein content in the cisplatin-treated group was significantly (*p < 0.05) decreased (28.9 ± 1 mg/mL) than the control group (68.07 ± 0.74 mg/mL). Berberis lyceum methanolic extract groups (MEB-200 and MEBL-400) have indicated an insignificant change in protein content as compared to the control.he simultaneous administration of the cisplatin and Berberis lyceum methanolic extract significantly increased protein content in contrast with the cisplatin-treated group. For group 5 and group 6, the respective increase in protein content 43.7 ± 1.4 mg/mL and 44.9 ± 1.2 mg/mL was noticed. The concurrent administration of the cisplatin and vitamin E also demonstrated a significant increase (47.2 ± 0.3 mg/mL) in protein content (Figure 2).

### Effect of Berberis lyceum methanolic extract on serum GST specific activity (µmol/min/mg)

This study showed that the GST specific activity in the doxorubicin-treated group was significantly (*p < 0.05) decreased (0.027 ± 0.006 µmol/min/mg) than the control group (0.068 ± 0.003 µmol/min/mg). The methanolic extract groups (MEBL-200 and MEBL-400) revealed an insignificant change in GST activity as compared to the control. The synchronous administration of the doxorubicin and Berberis lyceum methanolic extract significantly (*p < 0.05) enhanced the GST activity as compared to the doxorubicin-treated group. For group 5 and group 6, the increase in the GST activity was found to be 0.052 ± 0.001 µmol/min/mg and 0.056 ± 0.002 µmol/min/mg, respectively. The co-administration of the doxorubicin and vitamin E also exhibited a significant increase (0.057 ± 0.002 µmol/min/mg) in the GST activity as shown in Table 3.

**Table 3:** In vivo effect of Berberis lyceum methanolic extract on cardiac enzymes in doxorubicin-induced cardiotoxicity

GROUPS	GST (µM/min/mg protein)	MDA (µM/g tissue)	tGSH (mM/g tissue)	CAT (µM/g tissue)
**CONTROL**	0.068±0.003	14.2 ± 0.3	2.25±0.053	0.47± 0.12
**DOX**	0.027±0.006^#^	38.01 ± 0.2^#^	0.54±0.024^#^	0.29 ±0.13^#^
**MEBL200**	0.075±0.075	14.13 ± 0.30	2.28±0.04	0.52 ±0.145
**MEBL400**	0.08±0.005	13.93 ± 0.25	2.33±0.073	0.53 ± 0.096
**DOX+MEBL200**	0.052±0.001*	22.3 ± 0.46*	1.79±0.03*	0.37 ± 0.085*
**DOX+MEBL400**	0.056±0.002*	18.76 ± 0.3*	1.98±0.03*	0.48 ± 0.160*
**DOX+VIT-E**	0.057±0.002*	18.4 ± 0.34*	2.04 ±0.06*	0.45±0.068*

Analysis was conducted between doxorubicin and the control group by using an unpaired t-test, while analysis among all other groups was done by using one-way ANOVA followed by Dunnett's test. The values were expressed in Mean±SD (n=6)

* p<0.05 was considered significant.

The GST specific activity in the cisplatin-treated group was significantly (*p < 0.05) decreased (0.011 ± 0.01 µmol/min/mg) than the control group (0.058 ± 0.01 µmol/min/mg). The methanolic extract treated groups (MEBL-200 and MEBL-400) showed an insignificant change in the GST activity as compared to the control. The concurrent administration of the cisplatin and Berberis lyceum methanolic extract significantly enhanced GST activity as compared to the cisplatin-treated group. For group 5 and group 6, an increase in the GST activity 0.043 ± 0.01 µmol/min/mg and 0.049 ± 0.01 µmol/min/mg was estimated, respectively. The parallel administration of the cisplatin and vitamin E exhibited a marked increase (0.055 ± 0.01 µmol/min/mg) in the GST activity (Table 4).

**Table 4:** In vivo effect of Berberis lyceum methanolic extract on the brain enzymes in cisplatin-induced neurotoxicity

Groups	GST(µM/min/mg protein)	MDA (µM/g tissue)	tGSH (mM/g tissue)	CAT (µM/g tissue)
**Control**	0.058±0.01	88.4 ± 2.9	1.25±0.04	0.66 ± 0.02
**Cisplatin**	0.011±0.01#	183.9 ± 3.3^#^	0.48±0.06^#^	0.32±0.02^#^
**MEBL200**	0.06±0.01	84.4 ± 1.0	1.26±0.06	0.67 ±0.01
**MEBL400**	0.061±0.01	83.7 ± 1.0	1.29±0.03	0.68 ±0.02
**Cisplatin** **+MEBL200**	0.043±0.01*	93.4±0.6*	0.98±0.1*	0.59± 0.01*
**Cisplatin** **+MEBL400**	0.049±0.01*	91.4±0.4*	1.06±0.05*	0.61± 0.02*
**Cisplatin +VIT-E**	0.055±0.01*	89.0±2.6*	1.21±0.05*	0.64±0.03*

Analysis was conducted between doxorubicin and the control group by using an unpaired t-test, while analysis among all other groups was done by using one-way ANOVA followed by Dunnett's test. The values were expressed in Mean±SD (n=6)

* p<0.05 was considered significant.

### Effect of Berberis lyceum methanolic extract on serum tGSH (mmol/mg tissue) content

The current study outcomes have demonstrated that the tGSH level in the doxorubicin-treated group was significantly (*p < 0.05) decreased (0.54 ± 0.03 mmol/mg) than thcontrol group (2.25 ± 0.053 mmol/mg). Berberis lyceum methanolic extract groups (MEBL-200 and MEBL-400) showed an insignificant change in the tGSH level against the control. The concurrent administration of the doxorubicin with different doses of Berberis lyceum methanolic extract treated groups has significantly increased the tGSH level over the doxorubicin-treated group. For group 5 and group 6, the respective increase of 1.79 ± 0.003 mmol/mg and 1.98 ± 0.03 mmol/mg was seen and the results are shown in Table 3.

The tGSH level in the cisplatin-treated group was significantly (*p < 0.05) decreased (0.48 ± 0.06 mmol/mg) than control group (1.25 ± 0.04 mmol/mg). Berberis lyceum methanolic extract groups (MEBL-200 and MEBL-400) have indicated an insignificant change in the tGSH level as compared to control. The co-administration of the cisplatin with different doses of Berberis lyceum methanolic extract treated groups significantly increased the tGSH level against the cisplatin-treated group. For group 5 and group 6, the respective increase 0.98 ± 0.1 mmol/mg and 1.06 ± 0.05 mmol/mg tissue was estimated (Table 4).

### Effect of Berberis lyceum methanolic extract on serum CAT activity (µmol of H2 O2 consumed/min/mg protein)

The present investigation illustrated that the CAT activity in the doxorubicin-treated group was significantly (*p < 0.05) decreased (0.287 ± 0.13 µmol/min/mg) than the control group (0.470 ± 0.12 µmol/min/mg). Berberis lyceum methanolic extract groups (MEBL-200 and MEBL-400) showed an insignificant change in the CAT activity as compared to the control. The parallel administration of the doxorubicin and Berberis lyceum methanolic extract significantly (*p < 0.05) decreased the CAT activity in comparison to the doxorubicin-treated group. For groups 5 and 6, the respective decrease in CAT activity was 0.3691 ± 0.085 µmol/min/mg and 0.484 ± 0.160 µmol/min/mg. The concurrent administration of the doxorubicin with vitamin E also exhibited a significant decrease (0.447 ± 0.068 µmol/min/mg) in the CAT activity when compared with the doxorubicin-treated group (Table 3).

The CAT activity in the cisplatin-treated group was significantly (*p < 0.05) decreased (0.32 ± 0.02 µmol/min/mg) than the control group (0.66 ± 0.02 µmol/min/mg). Berberis lyceum methanolic extract groups (MEBL-200 and MEBL-400) demonstrated an insignificant change in CAT activity as compared to the control. The co-administration of the cisplatin and Berberis lyceum methanolic extract treated groups significantly decreased the CAT activity than the cisplatin-treated group. For group 5 and group 6, the respective decrease was 0.59 ± 0.01 µmol/min/mg and 0.61 ± 0.02 µmol/min/mg. The concomitant administration of the cisplatin with vitamin E produced a significant decrease (0.64 ± 0.03 µmol/min/mg) in the CAT activity as compared to the cisplatin-treated group (Table 4).

### Effect of Berberis lyceum methanolic extract on serum MDA content (µmol/g tissue)

The study results manifested that the MDA level in the doxorubicin-treated group was significantly (*p < 0.05) increased (38.01 ± 0.2 µmol/g) than the control group (14.2 ± 0.3 µmol/g). The methanolic extract groups (MEBL-200 and MEBL-400) showed an insignificant change in the MDA level as compared to the control. The concurrent administration of the doxorubicin with Berberis lyceum methanolic extract significantly decreased the MDA level in comparison to the doxorubicin treated group. For groups 5 and 6, the MDA level dropped to 22.3 µmol/g ± 0.46 and 18.76 ± 0.3 µmol/g, respectively. The synchronous administration of the doxorubicin with vitamin E also exhibited a significant decrease (18.4 ± 0.34 µmol/g) in the MDA level as compared to the doxorubicin-treated group (Table 3).

The MDA level in cisplatin-treated group was significantly (*p < 0.05) increased (183.9 ± 3.3 µmol/g) than control group (88.4 ± 2.9 µmol/g). The methanolic extract groups (MEBL-200 and MEBL-400) showed an insignificant change in the MDA level against the control. The co-administration of the cisplatin with Berberis lyceum methanolic extract significantly decreased the MDA level as compared to the cisplatin-treated group. For group 5 and group 6, the respective decrease was 93.4 ± 0.6 and 89 ± 2.6 and the results are presented in Table 4.

### Histo-pathological examination of heart and brain tissues

**Figure 3:**
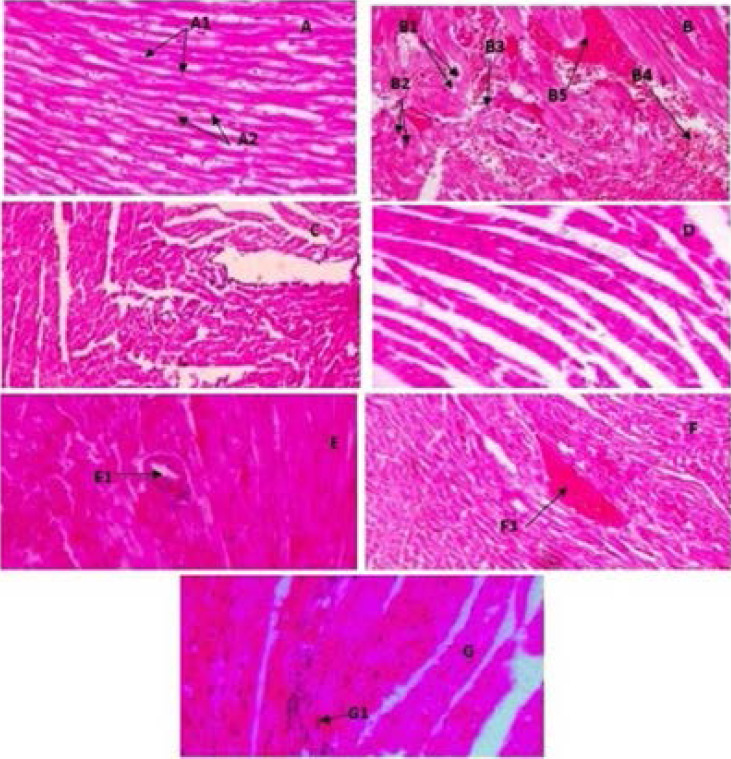
H&E stained light photomicrograph of rat heart treated by Berberis lyceum methanolic extract treated groups, 20X magnification. A: Control heart, A1: normal nuclei, A2: well-formed fibers, B: Doxorubicin (20 mg/kg BW) treated group, B1: vacuolization, B2: pyknotic nuclei, B3: vascular congestion, B4: coagulative necrosis, B5: hemorrhage, C: MEBL (200 mg/ kg BW), D: MEBL (400 mg/kg BW), E: DOX + MEBL (200 mg/kg BW), E1: vascular congestion, F: DOX + MEBL (400 mg/kg BW), F1: hemorrhage, G: DOX + vit-E (100 mg/kg BW), G1: pyknotic nucleu.

**Figure 4:**
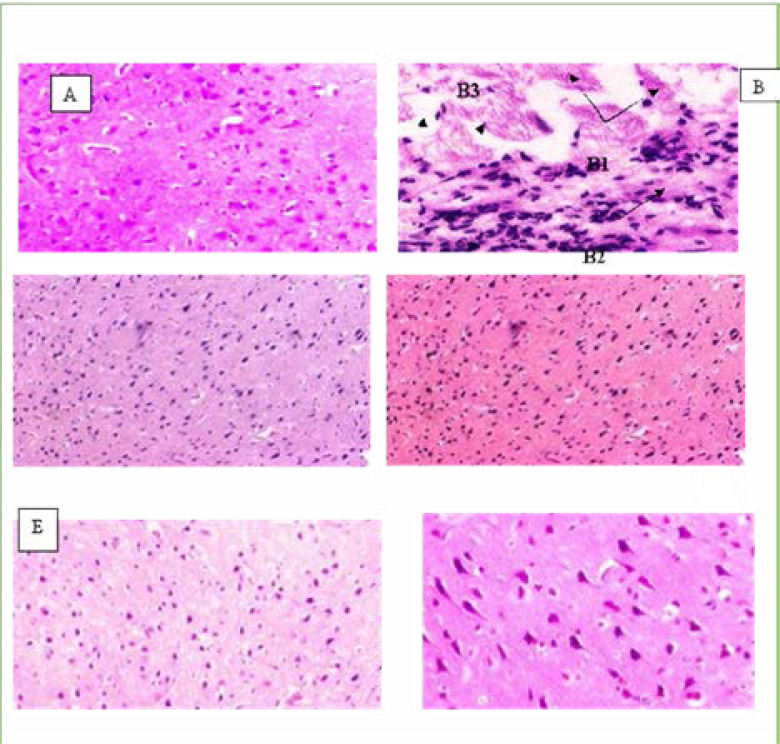
H&E stained light photomicrograph of rat brain treated by Berberis lyceum methanolic extract treated groups, 20X magnification. A: control brain, B: cisplatin (4 mg/kg BW), B1: liquefactive necrosis, B2: inflammatory cells, B3: vacuolization, C: MEBL (200 mg/kg BW), D: MEBL (400 mg/kg BW), D1: well formed nuclei, E: cisplatin + (MEBL 200 mg/kg BW), Fcisplatin + MEBL (400 mg/kg BW).

The histopathological examination of the control (normal) heart tissue showed the normal architecture of myocardiocytes like well-arranged fibers and nucleus with no vacuolization (Figure 3A). In the doxorubicin-treated group (20 mg/ kg), severely degenerated myocardiocytes were noticed with vacuolization of cytoplasm, loss of nuclei, sarcoplasm fragmentation, increased infiltration, coagulated necrosis of cardiac cells, and vascular congestion (Figure 3B). MEBL-200 mg/ kg and MEBL-400 mg/ kg treated groups exhibited histology similar to the control (Figures 3C and 3D). In Berberis lyceum methanolic extract (200 mg/kg) co-administered with the doxorubicin, the morphological observation of cardiac tissue showed an adequate degeneration of myocytes, moderate vacuolization of cytoplasm, pyknotic nuclei, less necrosis, and vascular congestion (Figure 3E). In Berberis lyceum methanolic extract (400 mg/kg) co-administered with the doxorubicin, morphological study of cardiac tissue illustrated a moderate degeneration of myocytes with less vacuolated cytoplasm and pyknotic nuclei. Further, remnants of necrosis along with little vascular congestion were present (Figure 3F). Vitamin E co-administered with doxorubicin manifested a moderate degeneration of myocytes with few cytoplasmic vacuolization and pyknotic nuclei. The remnants of necrosis and little congestion were also noticed. However, decreased infiltration of inflammatory cells was examined as well (Figure 3G). The histopathological analysis of the methanol extract at two doses (200 and 400 mg/ kg) showed improved morphology of the brain tissue, which was damaged due to the cisplatin administration as shown in Figures 4A and 4B. Moreover, the methanolic extract prevented the formation of pyknotic nuclei with the reduced vacuolization of neurons as illustrated in Figures 4C, 4D, 4E, and 4F, respectively. The study outcome has confirmed that both methanolic extract (200 and 400 mg/kg) have the potential to reverse the neurotoxicity caused by the anticancer drug.

## Discussion

Doxorubicin-induced cardiotoxicity and cisplatin-induced neurotoxicity are foremost challenges for the current chemotherapy practices and need to be addressed on an urgent basis. Doxorubicin is one of the widely prescribed broad-spectrum anti-cancer drugs. Doxorubicin causes severe cardiotoxicity that limits its clinical use.^32^ Doxorubicin induces cardiotoxicity due to the formation of free radicals and oxidative stress. Doxorubicin alters the serum enzymes level (ALT, AST, LDH and CK) and also produces marked morphological changes in cardiac tissue including necrosis, intravascular hemolysis, and congestion of the vessels.^33^ Likewise, cisplatin is widely used to treat cancer patients and induces neurotoxicity as a side effect.^34^ Many studies have demonstratedthat cisplatin causes brain damage via several mechanisms, i.e. increase lipid peroxidation, amplify the formation of free radicals and elevate the MDA level.^35^

Globally, medicinal plants are used to treat numerous acute and chronic illnesses because these are considered safe, effective, easily accessible, and inexpensive source of therapy.^36^,^37^ Berberis lyceum is a medicinal plant that is indigenous to India and Pakistan. It contains valuable bioactive chemicals and widely used to treat many disorders, i.e. diarrhea, inflammation, diabetes, gingivitis, jaundice, and ophthalmic disorders. It has strong antioxidant properties and quite beneficial in reducing the generation of free radicals. In this study, the pre-treatment of rats with the Berberis lyceum methanolic extract can mitigate doxorubicin and cisplatin-induced oxidative stress and prevent cardio and neurotoxicity. Since antioxidants can decrease oxidative stress by impeding the progression of reactive species production and lowering lipid peroxidation, this fact may be taken as a promising aspect by which Berberis lyceum defenses against unwanted side effects related to doxorubicin and cisplatin.^15^,^38^,^39^

In the present investigation, it is proved that the administration of Berberis lyceum root bark methanolic extract can improve or reverse the doxorubicin-induced and cisplatin-induced changes. Earlier studies have confirmed that the release of ALT, AST, LDH, CK, and CK-MB will increase from myocardial tissues with doxorubicin use. These enzymes are valuable tools to assess doxorubicin-induced myocardial toxicity. This study found that the tissue levels of ALT, AST, LDH, CK, and CK-MB were increased in doxorubicin-treated rats, and pre-treatment with Berberis lyceum root bark methanolic extract significantly decreased the levels of these enzymes.^40^ Further, doxorubicin and cisplatin considerably reduce the total protein content, GST specific activity, tGSH content, and CAT activity. In addition, doxorubicin and cisplatin appreciably (*p < 0.05) elevated the level of MDA. Berberis lyceum root bark methanolic extract significantly augmented the total protein content, GST specific activity, tGSH level and CAT activity when administered with anticancer drugs, and MDA level was considerably reduced.^41^

The histopathological examination of the heart and brain tissues revealed that doxorubicin and cisplatin both caused the damage of tissue morphology and resulted in cellular death. The histopathological evaluation of methanolic extract treated showed marked improvement in both organ tissues with moderate to mild degeneration. The results have demonstrated that the response of methanolic extract against anticancer drugs induced toxicity is dose-dependent as evident by improving enzymes level and tissue histopathology, i.e. mild protection is attained at a dose of 200 mg/kg while a significant defense against toxicity is achieved at an effective dose of 400 mg/kg.^42^,^43^ The plant phytochemicals are responsible for the protective effects against cardio and neurotoxicity induced by the chemotherapeutic agents. These phytochemicals can shield without decreasing the efficacy of synthetic drugs (doxorubicin and cisplatin) and are safe to use for a longer period.^44^,^45^ This study manifested, that Berberis lyceum is enriched with various phytochemicals such as flavonoids, polyphenols, saponins, and alkaloids. In a previous investigation, HPLC-UV characterization was performed on the plant root extract, and the presence of many chemical compounds (quercetin, chlorogenic acid, berberine, rutin, mandelic acid, and hydroxybenzoic acid) was reported. The chemical characterization of Berberis lyceum was also done by NMR and many important compounds were identified, such as berberine, β-sitosterol, 4-methyl, 7-hydroxy coumarin, and butyl-3-hydroxypropyl-phthalate. The generation of free radicals led to oxidative stress, which is a possible mechanism behind the induction of cardiotoxicity and neurotoxicity. These phytochemicals reduce oxidative stress by suppressing the generation of free radicals and hence protect against harmful effects of the anticancer drug.^46^ Additionally, doxorubicin and cisplatin are extensively metabolized in the liver and cause hepatotoxicity which is a serious side effect and frequently leads to stop therapy in cancer patients. Earlier experiments have proved that Berberis lyceum protects against hepatotoxicity by bringing the liver enzymes to their normal levels.^47^ Berberine which is an alkaloid present in this plant has the promising effect to reduce oxidative stress and proven beneficial against drug-induced liver toxicity.^48^ A study demonstrated that berberine possesses in vivo anti-inflammatory activity and inhibits the activity of activator protein 1, which triggers inflammatory cytokines such as interleukin-6. Many studies have documented that berberine is extremely target-specific with meager cytotoxicity on normal cells. It has tremendous ability to stop cell cycle at G1 phase specifically in tumor cells at lower concentration while arrest cell cycle at G2/M phase at higher concentration and stimulating apoptosis. The co-administration of berberine and anticancer drugs not only reduces cytotoxic effects of chemotherapeutic agents but also improves their therapeutic efficacy.^49^,^50^ However, further studies are required to translate Berberis lyceum protective mechanism, safety, and practical implications in clinical use.

## Conclusion

Berberis lyceum exhibited significant cardio and neuroprotection against anticancer drugs. The protective activity can be related to the plant antioxidant potential and its membrane-stabilizing effect by reducing lipid peroxidation. It is suggested that further research on Berberis lyceum Royle should be carried out to develop new and effective therapeutic agents to treat toxicity induced by anticancer drugs.

## Figures and Tables

**Table 5:** Histopathological scoring of cardiac tissue (Transverse sections) of rat in Doxorubicin induced cardiotoxicity

Groups	Vacuolization	Pyknotic nuclei	Vascular congestion	Coagulative necrosis	Hemorrhage
Control	-	-	-	-	-
DOX	+++	+++	+++	+++	+++
MEBL200	-	-	-	-	-
MEBL400	-	-	-	-	-
DOX+MEBL200	-	-	+	-	-
DOX+MEBL400	-	-	-	-	+
DOX+VIT-E	-	+	-	-	-

Sign - indicates absence of effect while +, ++ and +++ indicate mild, moderate and marked effect, respectively.

**Table 6:** Histopathological scoring of brain tissue (Transverse sections) of rat in Cisplatin induced neurotoxocity

Groups	Liquefactive necrosis	Inflammatory cells	Vacuolization
Control	−	−	−
Cisplatin	+++	+++	+++
MEBL200	−	−	−
MEBL400	−	−	−
Cisplatin +MEBL200	+	−	−
Cisplatin +MEBL400	+	−	−

Sign - indicates absence of effect while +, ++ and +++ indicate mild, moderate and marked effect, respectively.

## References

[1] Mollakhalili Meybodi N, Mortazavian AM, Bahadori Monfared A, Sohrabvandi S, Aghaei Meybodi F (2017). Phytochemicals in cancer prevention: a review of the evidence. Iranian J Cancer Prev.

[2] Shahbaz M, Kamran SH, Anwar R (2020). Amelioration of Bleomycin and Methotrexate-Induced Pulmonary Toxicity by Serratiopeptidase and Fisetin. Nutr Cancer.

[3] Bashash D, Zareii M, Safaroghli-Azar A, Omrani MD, Ghaffari SH (2017). Inhibition of telomerase using BIBR1532 enhances doxorubicin-induced apoptosis in pre-B acute lymphoblastic leukemia cells. Hematol.

[4] Biondo LA, Silveira LS, de Souza Teixeira AA, Neto JCR (2020). White adipose tissue and cancer: Impacts of doxorubicin and potential co-therapies. Immunometabolism.

[5] Arshad S, ur Rehman M, Abid F, Yasir S, Qayyum M, Ashiq K (2019). Current situation of breast cancer in Pakistan with the available interventions. Int J Biosci.

[6] Srivastava A, Amreddy N, Babu A, Panneerselvam J, Mehta M, Muralidharan R (2016). Nanosomes carrying doxorubicin exhibit potent anticancer activity against human lung cancer cells. Sci Rep.

[7] Shi Y, Moon M, Dawood S, McManus B, Liu P (2011). Mechanisms and management of doxorubicin cardiotoxicity. Herz.

[8] Govender J, Loos B, Marais E, Engelbrecht AM (2014). Mitochondrial catastrophe during doxorubicin-induced cardiotoxicity: a review of the protective role of melatonin. J Pineal Res.

[9] Mobaraki M, Faraji A, Zare M, Dolati P, Ataei M, Manshadi HD (2017). Molecular mechanisms of cardiotoxicity: a review on major side-effect of doxorubicin. Indian J Pharm Sci.

[10] Zhang Y-Y, Yi M, Huang Y-P (2017). Oxymatrine ameliorates doxorubicin-induced cardiotoxicity in rats. Cell Physiol Biochem.

[11] Schaupp CM, White CC, Merrill GF, Kavanagh TJ (2015). Metabolism of doxorubicin to the cardiotoxic metabolite doxorubicinol is increased in a mouse model of chronic glutathione deficiency: A potential role for carbonyl reductase 3. Chem Biol Interact.

[12] Aldossary SA (2019). Review on pharmacology of cisplatin: clinical use, toxicity and mechanism of resistance of cisplatin. Biomed Pharmacol J.

[13] Lomeli N, Di K, Czerniawski J, Guzowski JF, Bota DA (2017). Cisplatin-induced mitochondrial dysfunction is associated with impaired cognitive function in rats. Free Radic Biol Med.

[14] Avan A, Postma TJ, Ceresa C, Avan A, Cavaletti G, Giovannetti E (2015). Platinum-induced neurotoxicity and preventive strategies: past, present, and future. Oncologist.

[15] Ahmed S, Shuaib M, Ali K, Ali S, Hussain F (2017). Evaluation of different parts of Berberis lyceum and their biological activities: a review. Pure Appl Biol.

[16] Chand N, Durrani F, Qureshi M, Durrani Z (2007). Role of Berberis lycium in reducing serum cholesterol in broilers. Asian-Australasian J Anim Sci.

[17] Aslam H, Jehangir A, Naeem U (2015). Comparison of Hypoglycemic Activity of Berberis Lycium Royle Stem Bark and Glimepiride in Type 2 Diabetes. J Islamic In Med Coll.

[18] Singh A, Gupta M (2018). Phytochemical Investigation of Different Solvent Extracts Of Berberis Lycium Fruits. Rasayan J Chem.

[19] Anwar R, Sultan R, Batool F (2018). Ameliorating effect of Berberis lycium root bark extracts against cisplatin-induced nephropathy in rat. Bangladesh Journal of Pharmacology.

[20] Khan I, Najeebullah S, Ali M, Shinwari ZK (2016). Phytopharmacological and ethnomedicinal uses of the Genus Berberis (Berberidaceae): A review. Trop J Pharm Res.

[21] Lowry O (1951). Protein determination with the phenol reagent. J biol Chem.

[22] Besbes S, Blecker C, Deroanne C, Drira NE, Attia H (2004). Date seeds: chemical composition and characteristic profiles of the lipid fraction. Food Chem.

[23] Al-Hooti S, Sidhu SS, Gabazard H (1998). Chemical composition of seeds of date fruit cultivars of United Arab Emirates. J Food Sci Technol.

[24] Slinkard K, Singleton VL (1977). Total phenol analysis: automation and comparison with manual methods. Am J Enol Vitic.

[25] Chang CC, Yang MH, Wen HM, Chern JC (2002). Estimation of total flavonoid content in propolis by two complementary colorimetric methods. J Food Drug Ana.

[26] Hussain K, Ismail Z, Sadikun A, Ibrahim P (2008). Analysis of proteins, polysaccharides, glycosaponins contents of Piper sarmentosum Roxb. and anti-TB evaluation for bio- enhancing/interaction effects of leaf extracts with Isoniazid (INH). Nat Prod Rad.

[27] Anwar R, Hussain A, Ismail S, Mansor S (2012). In vitro effect of mitragynine on activity of drug metabolizing enzymes, n-demethylase and glutathione s-transferase in streptozotocin-induced diabetic rats. Pharmacologyonline.

[28] Sedlak J, Lindsay RH (1968). Estimation of total, protein-bound, and nonprotein sulfhydryl groups in tissue with Ellman's reagent. Anal Biochem.

[29] Sinha AK (1972). Colorimetric assay of catalase. Anal Biochem.

[30] Ohkawa H, Ohishi N, Yagi K (1979). Assay for lipid peroxides in animal tissues by thiobarbituric acid reaction. Anal Biochem.

[31] Thenmozhi AJ, Raja TRW, Janakiraman U, Manivasagam T (2015). Neuroprotective effect of hesperidin on aluminium chloride induced Alzheimer's disease in Wistar rats. Neurochem Res.

[32] Songbo M, Lang H, Xinyong C, Bin X, Ping Z, Liang S (2019). Oxidative stress injury in doxorubicin-induced cardiotoxicity. Toxicol Lett.

[33] Razavi BM, Karimi G (2016). Protective effect of silymarin against chemical-induced cardiotoxicity. Iranian J Basic Med Sci.

[34] dos Santos NAG, Ferreira RS, dos Santos AC (2020). Overview of cisplatin-induced neurotoxicity and ototoxicity, and the protective agents. Food Chem Toxicol.

[35] Turan M, Cayir A, Cetin N, Suleyman H, Turan IS, Tan H (2014). An investigation of the effect of thiamine pyrophosphate on cisplatin-induced oxidative stress and DNA damage in rat brain tissue compared with thiamine: thiamine and thiamine pyrophosphate effects on cisplatin neurotoxicity. Human Exp Toxicol.

[36] El-Dahiyat F, Rashrash M, Abuhamdah S, Farha RA (2020). Herbal medicines: a cross-sectional study to evaluate the prevalence and predictors of use among Jordanian adults. J Pharm Pol Pract.

[37] Latif A, Ashiq K, Qayyum M, Ashiq S, Ali E, Anwer I (2019). Phytochemical and pharmacological profile of the medicinal herb: Bryophyllum pinnatum. J Anim Plant Sci.

[38] Yu J, Wang C, Kong Q, Wu X, Lu J-J, Chen X (2018). Recent progress in doxorubicin-induced cardiotoxicity and protective potential of natural products. Phytomed.

[39] Latif A, Ashiq K, Ashiq S, Ali E, Anwer I, Qamar S (2020). Phytochemical analysis and in vitro investigation of anti-inflammatory and xanthine oxidase inhibition potential of root extracts of Bryophyllum pinnatum. J Anim Plant Sci.

[40] Sergazy S, Shulgau Z, Fedotovskikh G, Chulenbayeva L, Nurgozhina A, Nurgaziyev M (2020). Cardioprotective effect of grape polyphenol extract against doxorubicin induced cardiotoxicity. Sci Rep.

[36] Saadati H, Noroozzadeh S, Esmaeili H, Amirshahrokhi K, Shadman J, Niapour A (2020). The Neuroprotective Effect of Mesna on Cisplatin-Induced Neurotoxicity: Behavioral, Electrophysiological, and Molecular Studies. Neurotox Res.

[41] Afsar T, Razak S, Almajwal A, Shabbir M, Khan MR (2019). Evaluating the protective potency of Acacia hydaspica R. Parker on histological and biochemical changes induced by Cisplatin in the cardiac tissue of rats. BMC Complement Altern Med.

[42] Aziz MM, Abd El Fattah MA, Ahmed KA, Sayed HM (2020). Protective effects of Olmesartan and L-carnitine on doxorubicin-induced Cardiotoxicity in rats. Canadian J Physiol Pharmacol.

[43] Abushouk AI, Ismail A, Salem AMA, Afifi AM, Abdel-Daim MM (2017). Cardioprotective mechanisms of phytochemicals against doxorubicin-induced cardiotoxicity. Biomed Pharmacother.

[44] Hosseini A, Sahebkar A (2017). Reversal of doxorubicin-induced cardiotoxicity by using phytotherapy: a review. J Pharmacopuncture.

[45] Nazir N, Rahman A, Uddin F, Khan Khalil AA, Zahoor M, Nisar M, Ullah S, Ullah R, Ezzeldin E, Mostafa GA (2021). Quantitative Ethnomedicinal Status and Phytochemical Analysis of Berberis lyceum Royle. Agronomy.

[46] Sabir S, Tahir K, Rashid N, Naz S, Masood B, Shah MA, Sualeh M (2013). Phytochemical and antioxidant studies of Berberis lycium. Pak J Pharm Sci.

[47] Shah FA, Shakir S, Rafique S, Shuaib A (2021). Reduction of liver enzyme ALT by berberis lycium in acetaminophen induced hepatic damage in mice liver. Prof Med J.

[48] Chen X, Zhang Y, Zhu Z, Liu H, Guo H, Xiong C, Xie K, Zhang X, Su S (2016). Protective effect of berberine on doxorubicin induced acute hepatorenal toxicity in rats. Mol Med Rep.

[49] Jagetia GC, Baliga MS (2004). Effect of Alstonia scholaris in enhancing the anticancer activity of berberine in the Ehrlich ascites carcinoma-bearing mice. J Med Food.

[50] Iqbal MJ, Quispe C, Javed Z, Sadia H, Qadri QR, Raza S, Salehi B, Cruz-Martins N, Mohamed ZA, Jaafaru MS, Razis AF (2021). Nanotechnology-based strategies for berberine delivery system in cancer treatment: pulling strings to keep berberine in power. Front Mol Biosci.

